# Full Mouth Rehabilitation of a Rare Case of Hypodontia: A Case Report

**DOI:** 10.1155/crid/4126848

**Published:** 2025-10-28

**Authors:** Negin Aminianpour, Somayeh Zeighami

**Affiliations:** ^1^Department of Prosthodontics, School of Dentistry, Shahid Sadoughi University of Medical Sciences, Yazd, Iran; ^2^Dental Research Center, Dentistry Research Institute and Department of Prosthodontics, School of Dentistry, Tehran University of Medical Sciences, Tehran, Iran

**Keywords:** case report, deep bite, fixed partial denture, hypodontia, implant-supported dental prosthesis, mouth rehabilitation

## Abstract

Hypodontia with one or two permanent teeth is a common anomaly in dental clinics, while missing more than six is rare. Management of these cases is challenging because, other than the missing tooth problem, bone formation deficiency, imperfect vertical dimension of occlusion, and available tooth displacement will complicate the treatment procedure. Being in early youth and having high esthetic demands make these patients even more difficult to treat. Before starting the treatment, an exact diagnostic procedure is essential to pick the best treatment option. Also, to achieve predictable and satisfactory results, a predefined workflow is useful. The current study presents a step-by-step procedure from diagnosis to full mouth rehabilitation of an 18-year-old man who suffered from hypodontia with 21 missing permanent teeth. A diagnostic work-up was carried out and mocked up in the mouth. Dental implants were inserted, and printed provisional restorations were evaluated in terms of esthetics, phonetics, and vertical dimension. Implant-supported and tooth-supported full ceramic restorations were made based on the treatment plan. Mutually protected occlusion was designed and implemented. At the end, an occlusal splint was made to care for the restorations. The main take-away lesson from this case is that in the treatment of hypodontia patients, all treatment options should be evaluated, and a multidisciplinary approach should be followed.

## 1. Introduction

Hypodontia is one of the most frequent craniofacial anomalies [1]. It can occur both in deciduous and permanent dentition as a nonsyndromic isolated feature or a part of a genetic syndrome [1]. Universal prevalence of hypodontia is reportedly 6.4% with a significant difference by continent. Prevalence of hypodontia was reported to be the highest in Africa, followed by Europe, Asia, and Australia, with a lower prevalence in North America and Latin America. The prevalence of hypodontia is higher in women than in men [2]. The prevalence of hypodontia in the Iranian population was mentioned as 18.9% [3]. Missing one or two permanent teeth is common, while missing more than six is rare [1]. Ectodermal dysplasia syndrome is one of the etiologies of hypodontia. It occurs when two or more ectodermal structures fail to develop [4]. Treatment of hypodontia usually requires a multidisciplinary approach, encompassing surgical to prosthodontic procedures [5]. It should be noted that when implant treatment is needed for a patient with long-term edentulism, some special procedures may be necessary due to severe bone loss and tissue resorption. Also, in the absence of an ideal dentition and occlusion, orthodontic treatment may be required due to visible misalignment of teeth [6]. Additionally, missing teeth can lead to bone defects, making implant placement impossible. In such cases, alternative treatment plans such as FPD (fixed partial denture) should be considered. Fixed, removable, or a combination of fixed and removable partial/complete dentures may be required, depending on the extent of edentulism [7]. Recent advances in dental technology (digital dentistry) provide the opportunity to use stronger and more esthetically pleasing dental materials [8]. This study describes a multidisciplinary approach adopted for the management of an 18-year-old hypodontia patient with 21 missing permanent teeth, which is unique in its kind.

## 2. Case Presentation

### 2.1. Chief Complaint

An 18-year-old male patient was referred to the Department of Prosthodontics of the Faculty of Dentistry, Tehran University of Medical Sciences, Tehran, Iran, for treatment of hypodontia. The patient's chief complaint was an unesthetic appearance due to the extensive missing and misalignment of his permanent teeth.

### 2.2. History

The patient had no systemic disease. The patient did not report a history of missing deciduous teeth. He reported the missing of one and two permanent teeth in his two uncles. He had previously undergone fixed orthodontic treatment for diastema closure, midline correction, and intrusion of maxillary central incisors, which was not completely successful. Of the patient's primary teeth, #A, B, J, and K had amalgam restorations, while #K had undergone pulpotomy.

### 2.3. Examination

**Figure 1:**
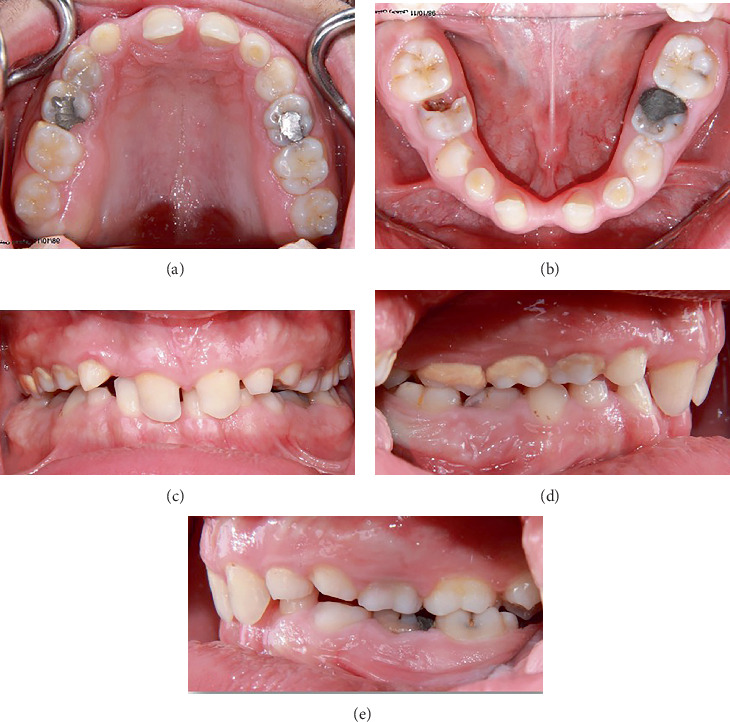
Pretreatment intraoral examination: (a) maxillary occlusal view, (b) mandibular occlusal view, (c) frontal view, (d) right lateral view, and (e) left lateral view.

**Figure 2:**
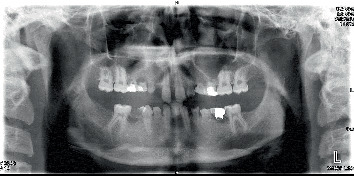
Pretreatment panoramic radiography.

The patient did not have abnormal facial asymmetry or lip incompetence. At rest, 3 mm of the patient's maxillary central incisors was visible. The mandibular anterior teeth were not visible at rest. The patient had a 2-mm midline shift toward the right side. His temporomandibular joints were asymptomatic. The patient had no parafunction. Intraoral examination revealed the absence of permanent teeth and retained primary teeth. The patient's remaining permanent teeth showed Class II Division 2 malocclusion with a deep bite and slight overjet (Figure 1). The buffer space showed more than 2-mm lateral movements, and the anterior guidance was steep. The patient had a moderate oral hygiene status (plaque index of 45%) and had no periodontal problems. In pronouncing the letters “f” and “w,” high-pressure contact of the central incisors on the lips was observed. Panoramic radiograph revealed the absence of Teeth #1, 4–7, 10–13, 16, 17, 18, 20–22, 24, 25, 28, 29, 31, and 32, and ectopic eruption of Tooth #27 in place of Tooth #28. Also, Tooth #19 was carious (Figure 2).

### 2.4. Diagnosis

Considering the absence of any anomaly in ectodermal structures of the patient, the diagnosis of ectodermal dysplasia was ruled out [4]. The patient gave written informed consent. There were no financial or cultural problems with the diagnosis and treatment procedures. At the first step, oral hygiene was instructed. After evaluation of the vertical dimension from the nose tip to the chin and based on phonetics, an interocclusal space of 6 mm was observed. Alginate impressions (Tropicalgin, Zhermack, Badia Polesine, Rovigo, Italy) were made from both dental arches, and centric relation (CR) was obtained using an anterior acrylic jig (Pattern Resin LS, GC America, Alsip, Illinois, United States) and wax bite plates (Cavex dental baseplates, Cavex Holland BV, Haarlem, Netherlands) with bilateral manipulation. An ear bow record (Face Bow AEE, Dentatus AB, Spanga, Sweden) was also obtained, and the settings of the nonarcon semiadjustable articulator (Dentatus ARH-Type, Dentatus AB, Spanga, Sweden) were set to the average. Light-cured restorative hybrid composite resin was applied on the mandibular left primary canine (Denfil, Vericom, South Korea) to reach the level of the lip corners. In the same treatment session, emergency treatment was performed for Tooth #19, which included caries removal and indirect pulp capping and filling with light-cure restorative glass ionomer (Fuji II LC Gold, GC, Tokyo, Japan). For compensating the 6-mm interocclusal space, the incisal pin of the articulator was opened 3 mm. Based on the composite index of the mandibular left primary canine, the maxillary anterior teeth were then waxed up and tried in. According to the amount of tooth shown, the incisal level of maxillary incisors remained intact. The direction of the occlusal plane (curves of Spee and Wilson) was determined by the Broadrick flag, and the wax-up of posterior teeth was completed and tried in. The interocclusal space at rest was 3 mm at this step of the treatment. It should be noted that after the final wax-up, the crown/root ratio of none of the teeth exceeded 1:1.

### 2.5. Periodontal and Endodontic Treatments

**Figure 3:**
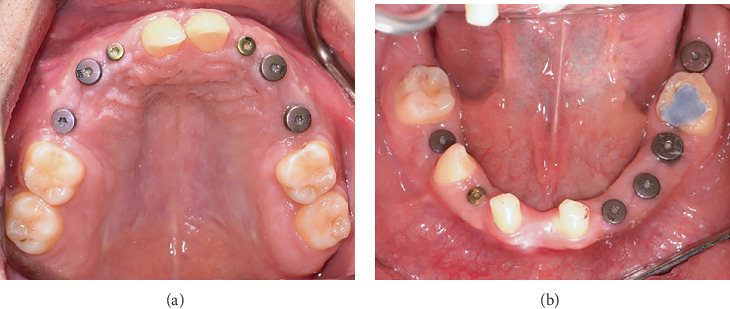
Intraoral view after implant insertion: (a) maxillary occlusal view and (b) mandibular occlusal view.

**Figure 4:**
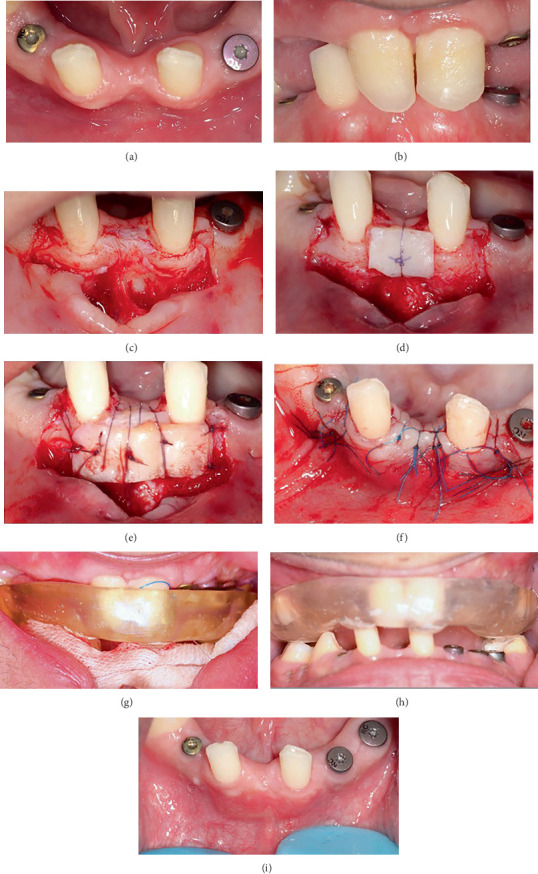
Preprosthetic surgery for the pontic area improvement: connective tissue grafting was performed using the pouch technique (a–i).

After reevaluation and improvement of the patient's oral hygiene status (plaque index = 21%), the patient underwent extraction of maxillary primary teeth and implant placement accompanied by open sinus lift surgery [9, 10]. On the right side of the maxilla, soft tissue was managed by a vascularized interpositional periosteal connective tissue flap [11, 12]. Details of the inserted implants are summarized in Table 1. All of the implants were from Straumann AG, Basel, Switzerland. To ensure leveling, a regular tissue-level implant was placed at the site of Tooth #20. Dental implantation at the site of Tooth #31 was not possible due to the placement on the vertical ramus and absence of sufficient soft tissue [13]. Implant insertion in the site of Teeth #24 and 25 was impractical due to severe bone deficiency; therefore, a full ceramic FPD with abutment Teeth #23 and 26 was proposed as the treatment plan for this area. To improve soft tissue status between Teeth #23 and 26 to create a suitable area for the pontic, connective tissue grafting was performed using the pouch technique [14] (Figure 3). To prevent traumatization of this region by the maxillary anterior teeth due to the patient's deep bite, an occlusal appliance was fabricated, and the patient used it full-time for 3 months. Finally, root canal treatment was performed for Tooth #19. Maxillary and mandibular arches after all periodontal and endodontic treatment are shown in Figure 4.

### 2.6. Prosthetic Treatment

After implant osseointegration (8 months after the placement of maxillary implants and 5 months after the placement of mandibular implants), a primary impression was taken to create a customized tray with VLC acrylic resin (Bisi-Tray, bisico, Bielefelder Dentalsilicone GmbH & Co. KG, Bielefeld, Germany). The prosthodontic treatment plan for each tooth is summarized in Table 2. The teeth were prepared with a round shoulder finish-line (by using a putty index to assess the amount of reduction). Dental implants were splinted using auto-polymerizing acrylic resin (Pattern Resin LS, GC America, Alsip, Illinois, United States), and the final impressions were made with the special tray with the two-phase one-step technique using injectable heavy-body (betasil VARIO HEAVY, Muller Omicron, Germany) and light-body (betasil VARIO LIGHT, Muller Omicron, Lindlar, Germany) addition silicone impression material. The CR record was also obtained using base and wax rims (Cavex Setup Regular Modeling Wax, Cavex Holland BV, Haarlem, Netherlands) by elastomeric bite registration material (Kristall Perfect A70, Muller Omicron, Lindlar, Germany). A HANAU spring bow record was also obtained, and the casts were mounted in a HANAU Wide-Vue arcon articulator.

For dental implants, screw-retained and for teeth, cement-retained provisional restorations using dental 3D printing resin color B1 (ARMA dental 3D printing resin, Temp Ultra, Turkey) and a dental 3D printer (Asiga Max UV, Irvine, California, United States), were made. Provisional restorations were adjusted to have a mutually protected occlusal scheme. Finalized provisional restorations were duplicated as casts, and a custom anterior guide table and a fossa guide were made using autopolymerizing acrylic resin. Using lateral interocclusal records made with wax, the condylar adjustments of the articulator were done as follows: 31° protrusive and 16° Bennett's angle on the left side and 33° protrusive and 16° Bennett's angle on the right side (the Hanau formula) [15].

**Figure 5:**
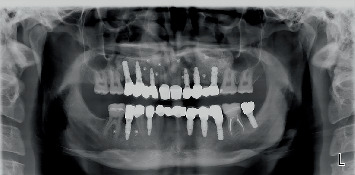
Posttreatment panoramic radiography.

**Figure 6:**
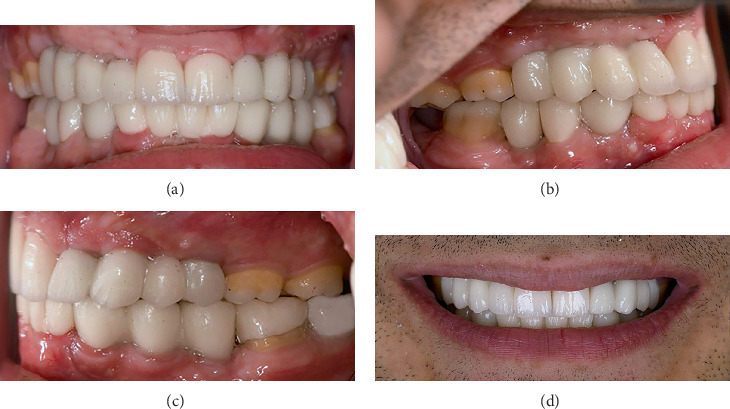
Full ceramic restoration delivery: (a) frontal view, (b) right lateral view, (c) left lateral view, and (d) smile view.

Next, the angulation, gingival height, and crown height space of dental implants were carefully evaluated according to the putty index, and final abutments were selected accordingly. Tooth and implant abutments were laboratory scanned, and full contour resin patterns were designed, printed, and tried-in. Next, the B1 tooth shade was selected by the patient. The zirconia frameworks were evaluated clinically and radiographically. The patient's specific fossa guide was used for correctly forming (veneering) the occlusal surface of restorations. Nonglazed restorations were tried-in to assess their function in centric and eccentric movements, phonetics, esthetics, and efficiency of the contacts. A mutually protected occlusal scheme was created. Finally, the restorations were glazed. A 30 N/cm torque was applied for regular implants, while a 20 N/cm torque was used for narrow implants. Tooth-supported crowns and bridges were cemented with glass ionomer cement (GC Fuji I, GI luting cement, GC Corp., Tokyo, Japan). Implant-supported crowns and bridges were cemented with zinc oxide eugenol temporary cement (Temp Bond, Kerr Corp., Orange, California, United States). The screw-cement-retained crown (#18), the endocrown (#19), and the occlusal veneer (#30) were cemented with dual-cure resin cement (TheraCem, BISCO Dental Products, Schaumburg, Illinois, United States) (Figure 5). To assess the presence of residual cement, ensure correct seating of restorations, and have a baseline radiograph for further evaluations in the future, full mouth periapical and panoramic radiographs (Figure 6) were obtained. The patient received oral hygiene instructions as well. To protect the restoration, a dual occlusal splint was fabricated. After delivery of the occlusal splint, follow-ups were scheduled.

### 2.7. Follow-Up and Outcomes

**Figure 7:**
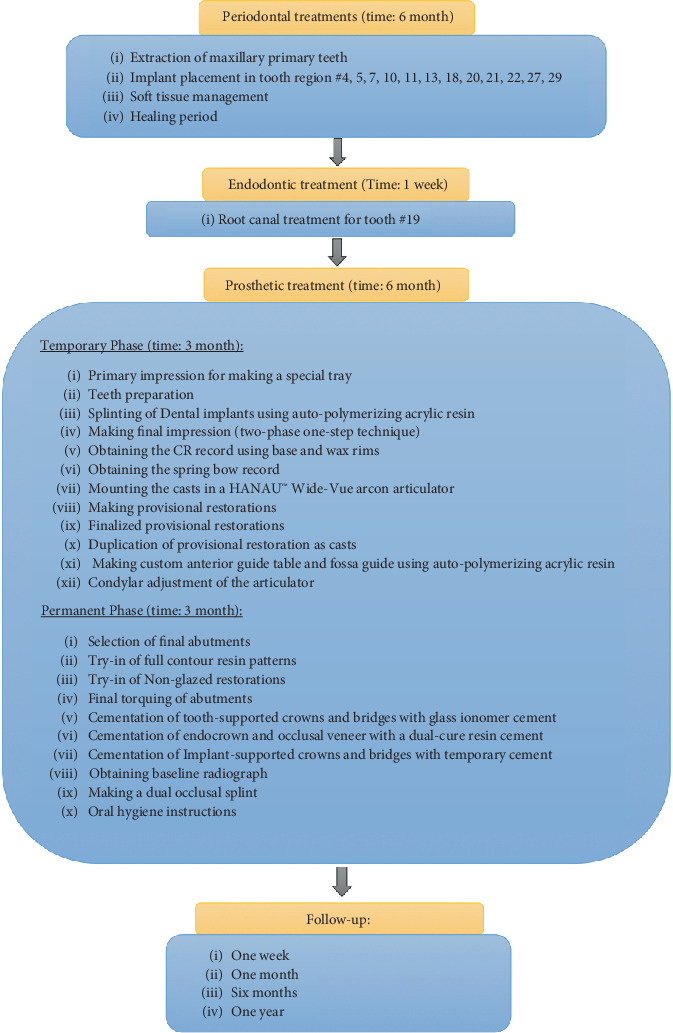
Treatment sequence flowchart of the patient.

Follow-ups were scheduled at 1 week, 1 month, 6 months, and 1 year after delivery. In each session, oral hygiene was emphasized, and the plaque index was evaluated. Occlusal stability was evaluated as well, and minor adjustments were done if needed. At the end of the 1-year follow-up, all teeth and implants were in a healthy situation from the perspective of prosthodontics and periodontics, and the patient was satisfied in terms of esthetics and function. To maintain the long-term stability of treatment and manage clinical outcomes, further follow-ups are needed. The treatment sequence flowchart is shown in Figure 7.

## 3. Discussion

In the present case, ectodermal dysplasia syndrome was ruled out since only one of the ectodermal structures, that is, teeth, had defects [4]. Several treatment options are available for the management of partial edentulism of permanent dentition: implant-supported FPDs, tooth-supported FPDs, tooth-supported removable partial dentures, and implant-supported removable partial dentures. The treatment of choice depends on factors such as the number of missing teeth, need for lip support, presence of posterior abutments, and the patient's financial status [13, 16]. In the present case, the abovementioned first and second treatment options were selected based on the patient's intraoral conditions, optimal prognosis of the remaining teeth, patient's age, financial status, and no need for additional labial support. A systematic review pointed to the correlation of missing permanent teeth and problems such as infraocclusion of the primary first molar and ectopic tooth eruption [17]. In this patient, ideal orthodontic treatment was not possible due to the absence of permanent teeth and a lack of sufficient anchorage. Maxillary central incisors required correction due to problems such as the presence of a diastema, midline deviation, and rotation. Since orthodontic treatment would not be successful even by insertion of miniscrews, restorative treatments (full crowns) were considered instead. Lost vertical dimension of occlusion (VDO) is one of the clinical appearances in hypodontia patients that affects patient function and esthetics significantly. Restoring lost VDO in such a case is indicated up to 5 mm and on condition of establishment of occlusal stability without detrimental consequences. Since the patient had a 6-mm interocclusal space, the VDO was increased by 3 mm [18]. Due to the relatively long period of edentulism, the maxillary sinus had become pneumatized, and surgical hard tissue management was required in some areas as for the replacement of Teeth #4 and 13. In the maxilla, open sinus lift, leveling with the adjacent implant at the site of Tooth #4, and bone powder (allograft) plus membrane application at the site of Tooth #13 were performed [19]. Implant placement at the site of Tooth #18 had a lot of advantages. The main advantage is that the implant-supported crown makes a more effective occlusal force; another advantage is preventing the eruption of the opposing tooth (#15). Furthermore, it prevents the movement of Tooth #19 and the opening of the contact with the implant-supported crown at the site of Tooth #20. Tooth #18 was restored with a screw–cement-retained restoration due to limited interarch space and concerns about access to the removal of excess cement. Screw–cement-retained restoration is a type of restoration that uses the advantages of both cement and screw-retained restorations. A prefabricated abutment was cemented to a monolithic zirconia restoration using dual-cure resin cement and then screwed onto the implant. Then, the screw access hole was filled with Teflon tape and covered by composite resin with the same color as the crown. Screw-retained restoration is recommended in limited interarch space (less than 4 mm) and the necessity for retrievability. Likewise, cement-retained restoration in the case of inclined implants is suggested [20]. Also, due to the absence of sufficient attached gingiva at the vertical ramus, implant placement at the site of Tooth #31 was not performed. Thus, a mesial contact was created for Tooth #2 with the distal area of occlusally veneered Tooth #30 to prevent overeruption.

In deep bite cases, soft tissue grafts placed in the anterior mandible (pouch technique) are at risk of traumatization by the opposing occlusion. To prevent this, a full-time dual occlusal appliance was designed for the patient, which was in contact with all teeth in CR. It not only protected the soft tissue but also further stabilized the remaining teeth.

In the final occlusion, considering the ectopic eruption of the canine at the site of Tooth #28, an ideal canine tooth for a true canine guide was not present. Moreover, a narrow implant was placed at the site of Tooth #27 to replace the canine and tooth-supported FPD for the replacement of mandibular central incisors, offering anterior group function to involve a higher number of anterior teeth in lateral excursions. Anterior group function could include a canine, a lateral, and both central incisors. In some cases, only the incisors contact in lateral excursions [6]. Considering the presence of multiple implant-supported restorations, a dual occlusal splint was used to protect them [13].

In a meta-analysis, the survival rate of partial-arch all-ceramic implant-supported fixed dental prostheses was estimated to be 98.3% after 5 years. The survival rate of implants supporting partial-arch all-ceramic implant-supported fixed dental prostheses was calculated to be 98.5% after 5 years. However, chipping of the veneering ceramic was a common outcome and was estimated to be 22.8% in 5 years, which is clinically unacceptable. One screw-loosening and 11 decementations were reported in 540 implant-supported fixed dental prostheses [21]. In a meta-analysis study, survival and success of endocrowns after 5 years were estimated to be 91.4% and 77.7%, respectively, while for conventional crowns they were 98.3% and 94%, respectively [22]. The success rate of occlusal veneer restorations was reported to be 92.6%, which was the same pulp survival rate [23].

The strength of this case was that the patient was young, fully motivated, and cooperative and also had no financial or cultural problems.

The limitation of this case report was the lack of a quantitative assessment of patient satisfaction and functional improvement after treatment. Also, reports of long-term follow-ups in future articles could be beneficial to present the survival rate of implants, possible biomechanical complications, and how to manage them.

## 4. Conclusion

Within the limitations of this case presentation, the following was concluded:

1. The primary take-away lesson of this case report is that proper management of patients with extensive hypodontia often requires a multidisciplinary approach.
2. In full mouth oral rehabilitation of such cases, the treatment plan and the order and sequence of the procedures should first be determined through diagnostic phases.
3. It should be noted that in cases with long-term edentulism, the treatment is often more complex, requiring more advanced procedures.
4. Selective (nonemergency) treatments should be postponed until optimal oral hygiene is established.
5. Regular follow-ups are also required to control treatment stability in such cases.

## Figures and Tables

**Table 1:** Inserted implant specifications.

**Tooth number (universal numbering system)**	**Connection type**	**Implant specifications**
4, 13, 18	Regular CrossFit (RC)	Bone level implants, SLActive, BL 4.1∗10 RC
5, 11, 21, 22, 29	Regular CrossFit (RC)	Bone level tapered implants, SLActive, BLT 4.1∗10 RC
7, 10, 27	Narrow CrossFit (NC)	Bone level tapered implants, SLActive, BLT 3.3∗10 NC
20	Regular neck (RN)	Standard implants, SLActive, S 4.1∗10 RN

**Table 2:** Treatment for each tooth.

**Tooth number (universal numbering system)**	**Treatment plan**	**Materials**	**Manufacturers**
2, 3, 14, 15	No treatment	—	—
4, 5, 6, 7	Implant-supported FPD with pontic #6 (cement-retained)	Core (Zolid Bion)–veneer (CERABIEN ZR)	Amann Girrbach AG, Austria–Kuraray Noritake, Japan
8, 9	Full crown	Core (Zolid Bion)–veneer (CERABIEN ZR)	Amann Girrbach AG, Austria–Kuraray Noritake, Japan
10, 11, 12, 13	Implant-supported FPD with pontic #12	Core (Zolid Bion)–veneer (CERABIEN ZR)	Amann Girrbach AG, Austria–Kuraray Noritake, Japan
18	Implant-supported crown (screw–cement retained)	Monolithic zirconia (Zolid Bion)	Amann Girrbach AG, Austria
19	Endocrown	IPS e.max Press	Ivoclar Vivadent GmbH, Liechtenstein
20, 21, 22	Implant-supported crown	Core (Zolid Bion)–veneer (CERABIEN ZR)	Amann Girrbach AG, Austria–Kuraray Noritake, Japan
23, 24, 25, 26	Tooth-supported FPD with pontic #24, 25	Core (Zolid Bion)–veneer (CERABIEN ZR)	Amann Girrbach AG, Austria–Kuraray Noritake, Japan
27, 29	Implant-supported crown	Core (Zolid Bion)–veneer (CERABIEN ZR)	Amann Girrbach AG, Austria–Kuraray Noritake, Japan
28	Full crown	Core (Zolid Bion)–veneer (CERABIEN ZR)	Amann Girrbach AG, Austria–Kuraray Noritake, Japan
30	Occlusal veneer	IPS e.max Press	Ivoclar Vivadent GmbH, Liechtenstein

## Data Availability

The data used to support the findings of this study were supplied by Somayeh Zeighami under license, and the data will be available on request. Requests for access to these data should be made to Somayeh Zeighami.

## Patient Perspective

I believe I received the best treatment in a reasonable amount of time. I am completely satisfied with the treatment process and its results, both esthetically and functionally. I hope that by maintaining proper hygiene and having regular follow-up sessions, I can extend the life of the treatment.
